# Medical and Long-Term Care Costs of Alzheimer’s Disease in Japan According to Clinical Dementia Rating Scores: The LIFE Study

**DOI:** 10.31662/jmaj.2025-0078

**Published:** 2025-11-28

**Authors:** Sung-a Kim, Futoshi Oda, Megumi Maeda, Fumiko Murata, Haruhisa Fukuda

**Affiliations:** 1 Department of Health Care Administration and Management, Graduate School of Medical Sciences, Kyushu University, Fukuoka, Japan

**Keywords:** Alzheimer’s disease, Medical costs, Long-term care costs, claims data

## Introduction

Alzheimer’s disease (AD) is a neurodegenerative disease associated with high morbidity and mortality ^(1)^. As AD progresses, it imposes increasingly heavy economic and psychological burdens on patients, their families, and society ^(2), (3), (4)^.

AD care involves a wide variety of long-term care (LTC) services that correspond to different disease stages, but many people with AD rely on family or unpaid caregivers ^(5)^. Although accurate estimates of AD’s economic burden are crucial for healthcare policymaking and cost-effectiveness research, few studies in Japan have comprehensively quantified the medical and LTC costs of AD patients according to disease severity. There is a need to establish standardized parameters for such cost estimates, which are essential for economic evaluations of AD-specific treatments and provide a fundamental basis to support the optimal distribution of health insurance and societal resources. As new drugs specifically targeting AD (e.g., lecanemab) have been introduced in recent years, there is growing interest in their cost-effectiveness.

This study aimed to estimate the severity-specific medical and LTC costs of older Japanese adults diagnosed with AD.

## Methods

### Study design and participants

This retrospective cohort study used data from the Longevity Improvement & Fair Evidence Study, a database project that collects medical, LTC, health checkup, and administrative data from Japanese municipalities ^(6)^. Our study database comprised medical and LTC claims data from 12 municipalities between April 2021 and March 2022. In addition to insurance-covered medical and LTC services, the data included recorded diagnoses based on International Classification of Diseases, 10th Revision (ICD-10) codes. Under Japan’s LTC insurance system, enrollees are assigned a care needs level at the time of care needs certification or renewal. Thereafter, certified care needs levels are indicated in LTC claims data whenever enrollees use LTC services. Although the care needs certification survey collects information on the Daily Life Independence Level of Older Persons with Dementia, this information is not included in LTC claims data. Given that we did not have access to care needs certification survey data for this study, our analysis used the readily available care needs levels in LTC claims data.

We identified older adults aged ≥65 years who had a recorded diagnosis of AD (ICD-10 codes: F00 or G30) (Supplementary Table 1), certified care needs, and were using any LTC services in April 2021; cases with suspected diagnoses were excluded. Individuals who died or received public assistance during the observation period were excluded. Patients receiving public assistance may overuse services because of low out-of-pocket payments, which could overestimate the overall mean costs for AD patients.

### Medical costs and LTC costs

We determined each participant’s monthly medical and LTC costs for the study period and calculated mean monthly costs by care needs level. We created a dataset that pooled monthly medical and LTC costs for each care needs level across each participants’ 12 months of data. If a participant’s certified care needs levels changed during the study period, monthly costs for each level were aggregated separately. Mean monthly costs were multiplied by 12 to calculate annual medical and LTC costs. This approach accounted for patient-level changes in care needs level over the 12-month period. Medical and LTC costs included all expenditures incurred during the study period and were not limited to AD treatments. Medical costs included expenditures for outpatient visits, medications, inpatient care, and medical/surgical procedures. LTC costs were categorized into “home LTC costs” (in-home LTC support, in-home services, and community-based services) and “facility LTC costs” (facility-based services) (Supplementary Table 2). All insurance-covered services were classified into one of these categories, and none were excluded from analysis. We calculated each participant’s home LTC costs and facility LTC costs as the weighted mean of expenditures for the corresponding services.

When patient-level costs are used in cost-effectiveness models, the appropriate denominator depends on the analytical perspective: when estimating the mean general cost per patient, the denominator should include all patients regardless of medical or LTC service use; when estimating the mean cost per patient within a group that uses a specific service, the denominator should be limited to service users. This study estimated costs using both denominators.

### Severity-specific costs

Although the study database did not include information on dementia scales, such as Clinical Dementia Rating (CDR) scores, each participant’s care needs level could be determined from the LTC claims data. Therefore, we used care needs levels as an initial proxy for AD severity. The distribution of individuals with certified care needs levels was analyzed according to their corresponding CDR scores (Supplementary Table 3) based on a previously reported relative distribution of these two scales in a Japanese population ^(7)^. CDR scores were classified into five categories ^(8)^: CDR0 (no impairment), CDR0.5 (mild cognitive impairment due to AD), CDR1.0 (mild), CDR2 (moderate), and CDR3 (severe). The weighted mean annual medical and LTC costs were initially calculated according to care needs level. Next, we converted these to the weighted mean annual medical and LTC costs for each CDR score using the aforementioned distribution.

## Results

The study cohort comprised 20,449 individuals (Table 1). The proportion of men decreased as care needs level increased (Support Level 1: 36.0%, LTC Level 5: 15.3%). The mean age was 86.1 years (standard deviation, 6.1).

**Table 1.** Participant Characteristics According to Care Needs Level^a^.

Characteristics		Total (N = 20,449)	Support Level 1 (n = 764)	Support Level 2 (n = 645)	LTC Level 1 (n = 6,623)	LTC Level 2 (n = 4,039)	LTC Level 3 (n = 3,607)	LTC Level 4 (n = 2,823)	LTC Level 5 (n = 1,948)
Sex	Men	4,844 (23.7)	275 (36.0)	168 (26.0)	1,696 (25.6)	1,054 (26.1)	816 (22.6)	536 (19.0)	299 (15.3)
	Women	15,605 (76.3)	489 (64.0)	477 (74.0)	4,927 (74.4)	2,985 (73.9)	2,791 (77.4)	2,287 (81.0)	1,649 (84.7)
Age	Mean years [SD]	86.1 [6.1]	84.5 [5.4]	85.5 [5.7]	85.2 [5.7]	86.5 [5.8]	86.7 [6.2]	87.3 [6.5]	86.7 [6.8]
	65-69 years	162 (0.8)	6 (0.8)	8 (1.2)	45 (0.7)	26 (0.6)	21 (0.6)	29 (1.0)	27 (1.4)
	70-74 years	597 (2.9)	28 (3.7)	18 (2.8)	211 (3.2)	89 (2.2)	111 (18.6)	70 (2.5)	70 (3.6)
	75-79 years	2,172 (10.6)	96 (12.6)	74 (11.5)	842 (12.7)	358 (8.9)	352 (9.8)	242 (8.6)	208 (10.7)
	80-84 years	4,583 (22.4)	235 (30.7)	153 (23.7)	1,747 (26.4)	873 (21.6)	701 (19.4)	499 (17.7)	375 (19.2)
	85-89 years	6,822 (33.4)	276 (36.1)	244 (37.8)	2,248 (33.9)	1,439 (35.6)	1,177 (32.6)	879 (31.1)	559 (28.7)
	≥90 years	6,113 (29.9)	123 (16.1)	148 (23.0)	1,530 (23.1)	1,254 (31.0)	1,245 (34.5)	1,104 (39.1)	709 (36.4)

Values are presented as n (%) unless indicated otherwise. LTC: long-term care; SD: standard deviation. ^a^Care needs levels in April 2021 were used.

Table 2 shows the mean annual medical and LTC costs according to estimated CDR score (the corresponding costs by care needs level are presented in Supplementary Table 4). When the denominator was limited to service users, the analysis showed that medical costs, home LTC costs, and facility LTC costs increased with higher CDR scores. Both home LTC costs and facility LTC costs demonstrated more pronounced increases than medical costs. Facility LTC costs were the highest for all CDR scores, peaking at 3.50 million yen (3.7 times the medical costs) for CDR3. Similarly, home LTC costs increased with higher disease severity, reaching 1.66 million yen (1.7 times the medical costs) for CDR3. While medical costs also increased with higher disease severity, these increases were not as substantial as those of LTC costs. When the denominator included all participants, the analysis again showed that medical costs, home LTC costs, and facility LTC costs increased with higher CDR scores. Home LTC costs reached 2.13 million yen (2.3 times the medical costs) for CDR3. Facility LTC costs rose from 7,437 yen (CDR0) to 1.05 million yen (CDR3), while medical costs rose from 53,586 yen (CDR0) to 920,929 yen (CDR3).

**Table 2.** Estimates of Mean Annual Medical Costs and LTC Costs in Clinically Diagnosed AD Patients According to CDR Score.

Costs	CDR0	CDR0.5	CDR1	CDR2	CDR3
Denominator limited to participants who used each corresponding service					
Medical costs	56,283	203,495	540,446	827,059	955,408
Home LTC costs	38,717	171,230	591,363	1,116,586	1,662,994
Facility LTC costs	78,745	409,470	1,580,673	2,915,462	3,498,879
					
Denominator included all participants					
Medical costs	53,586	193,474	512,677	785,852	920,929
Home LTC costs	67,152	295,535	1,004,862	1,780,112	2,127,550
Facility LTC costs	7,437	38,568	162,808	422,484	1,046,048

Costs are presented in Japanese yen. AD: Alzheimer’s disease; CDR: Clinical Dementia Rating; LTC: long-term care.

## Discussion

This study estimated medical and LTC costs for 20,449 older adults with AD in Japan across CDR scores. These estimates provide insight into the comprehensive economic burden by disease severity and may inform AD-related healthcare policymaking and cost-effectiveness research.

Our results showed that medical and LTC costs increase with higher CDR scores. In our sample, greater care needs were accompanied by higher hospitalization costs but not by a greater number of Charlson comorbidities (data not shown). This pattern suggests that the observed increases in medical and LTC costs with higher CDR scores reflect greater need for these services due to AD itself.

Home LTC costs were higher when the denominator included all participants versus only service users. This likely reflects large month-to-month variation in the numbers of service users across the three home LTC service types (in-home LTC support, in-home services, and community-based services), with particularly few community-based service users. Given that the weighted mean costs reflect monthly changes in the number of users, only service users as the denominator likely yielded lower annual cost estimates.

Home and facility LTC costs increased more than medical costs as CDR scores rose. Thus, the severity-specific economic burden for patients with AD cannot be fully captured by medical costs alone; LTC costs represents the larger contributor. Future economic studies on AD should adopt a comprehensive perspective that encompasses both medical and LTC costs. Furthermore, our findings reaffirm the importance of early interventions to control disease progression, such as preventive care and medical treatments starting from CDR0 (no impairment).

Although previous studies from various countries have reported that the healthcare costs of patients with AD increase with higher severity ^(9), (10), (11), (12), (13), (14), (15)^, few have included LTC costs ^(9), (15)^. A Spanish study of 343 patients with AD found that higher CDR scores were associated with larger increases in social care costs than in direct medical costs ^(9)^, while a Taiwanese study of 231 patients with dementia showed that higher CDR scores were associated with a small decrease in medical costs but a large increase in social care costs ^(15)^. Both studies underscore the importance of social care (i.e., LTC) costs among people with AD, aligning with our findings.

This study has several limitations. First, the study was conducted using data from 12 municipalities, and it may not be indicative of the overall Japanese population. Second, AD cases were identified using only ICD-10 codes, and we could not access confirmatory diagnostic information such as amyloid-β pathology. Third, our cost estimates were for all medical and LTC services used during the study period and included costs attributable to diseases other than AD. Fourth, our study focused on clinically diagnosed AD patients and did not include individuals with mild cognitive impairment who would likely have been in the CDR0-CDR1 groups. Due to this potential selection bias, our study may have overestimated the costs for patients with lower CDR scores, limiting their generalizability. Fifth, our study used a published distribution of CDR scores and care needs levels derived from a younger population (77.6 years) with fewer women (56%) ^(7)^. These differences may affect the suitability of directly applying this distribution to our study population. Moreover, the lack of age- and sex-distributed data precluded analyses that adjust for these parameters. However, that report provided the only distribution between these two scales in Japan and, as such, was used as the “best available data” for our analysis.

Our estimates of severity-specific medical and LTC costs of older adults with AD contribute to our understanding of their economic burden in Japan and provide cost parameters that could be used for future cost-effectiveness assessments of AD treatments.

## Article Information

### Author Contributions

Conceptualization and design: Sung-a Kim and Haruhisa Fukuda. Data acquisition: Megumi Maeda, Fumiko Murata, and Haruhisa Fukuda. Data analysis and manuscript drafting: Sung-a Kim. Analysis and interpretation of data: Futoshi Oda, Megumi Maeda, Fumiko Murata, and Haruhisa Fukuda. Supervision: Haruhisa Fukuda. Critical review for important intellectual content: all authors. Final approval of the version to be published: all authors.

### Conflicts of Interest

Haruhisa Fukuda received grants from Eisai Co., Ltd. and Biogen Inc.

### Approval Code Issued by the Institutional Review Board (IRB) and the Name of the Institution

The study was approved by the Kyushu University Institutional Review Board for Clinical Research (No. 22114-09).

## Supplement

Supplementary Material
