# Supplementary material for: Medical and Long-Term Care Costs of Alzheimer’s Disease in Japan According to Clinical Dementia Rating Scores: The LIFE Study
Source: JMA J. 2025 Nov 28;9(1):381–4. doi: 10.31662/jmaj.2025-0078 (PMC12888962; doi:10.31662/jmaj.2025-0078)
Supplement: Supplementary Material [file 2433-3298-9-1-0381-s001.pdf]

Supplementary Table 1. ICD-10 codes used for defining Alzheimer's disease

	ICD-10 Codes
Alzheimer's disease	F00, G30

ICD-10, International Classification of Diseases, 10th Revision.

Supplementary Table 2. Classification of LTC services using Japanese LTC Insurance procedural codes

Service type	Codes
In-home LTC support	43, 46
In-home services	11, 12, 13, 14, 15, 16, 17, 21, 22, 23, 24, 25, 26, 27, 2A, 2B, 31, 33, 34, 41, 42, 44, 45, 61, 62, 63, 64, 66, 67, A1, A2, A3, A4, A5, A6, A7, A8, A9, AA, AB, AC, AD, AE
Community-based services	28, 32, 36, 37, 38, 39, 54, 68, 69, 71, 72, 73, 74, 75, 76, 77, 78, 79
Facility-based services	51, 52, 53, 55, 59

LTC, long-term care.

Supplementary Table 3. Distribution of the proportion of individuals with certified care needs levels according to CDR score

		No application/Independent	Support Level 1	Support Level 2	LTC Level 1	LTC Level 2	LTC Level 3	LTC Level 4	LTC Level 5
CDR	0	0.936	0.023	0.019	0.012	0.005	0.003	0.002	0.001
	0.5	0.772	0.058	0.052	0.056	0.033	0.016	0.007	0.005
	1	0.411	0.072	0.066	0.17	0.158	0.075	0.04	0.009
	2	0.128	0.038	0.014	0.213	0.265	0.194	0.114	0.033
	3	0.082	0.003	0.003	0.039	0.092	0.187	0.321	0.272

CDR, Clinical Dementia Rating; LTC, long-term care.

Source: Asada T. Dementia Prevalence in Urban Areas and Measures Against the Impairment of Life Function by Dementia. MHLW Comprehensive Research Project on Dementia Measures. 2013.

Supplementary Table 4. Estimates of mean annual medical costs and LTC costs in clinically diagnosed AD patients according to care needs level

	Support Level 1	Support Level 2	LTC Level 1	LTC Level 2	LTC Level 3	LTC Level 4	LTC Level 5
**Denominator limited to participants who used each corresponding service**							
Medical costs	747,724	1,007,741	823,345	978,080	989,497	1,053,958	1,116,727
Home LTC costs	242,611	380,727	856,235	1,177,424	1,609,126	1,953,237	2,171,200
Facility LTC costs			3,402,992	3,439,000	3,580,256	3,903,936	4,136,987
**Denominator included all participants**							
Medical costs	707,232	971,669	773,267	919,508	919,508	1,016,933	1,089,272
Home LTC costs	426,174	705,843	1,671,066	2,235,526	2,373,917	2,294,868	2,468,830
Facility LTC costs			50,235	130,171	865,076	1,381,340	1,567,666

Costs are presented in Japanese yen.

AD, Alzheimer’s disease; LTC, long-term care.
